# Synchronous bilateral breast cancer in a male

**DOI:** 10.3332/ecancer.2013.377

**Published:** 2013-12-04

**Authors:** María Caridad Rubio Hernández, Yenia Ivet Díaz Prado, Suanly Rodríguez Pérez, Ronald Rodríguez Díaz, Zaili Gutiérrez Aleaga

**Affiliations:** 1 Mastology Department, Cuban National Institute of Oncology and Radiobiology, 29 y F Vedado, Plaza, La Habana 10400, Cuba; 2 Anatomical Pathology Department, Cuban National Institute of Oncology and Radiobiology, 29 y F Vedado, Plaza, La Habana 10400, Cuba

**Keywords:** male breast cancer, synchronous, bilateral, multifocal

## Abstract

Male breast cancer, which represents only 1% of all breast cancers, is occasionally associated with a family history of breast cancer. Sporadic male breast cancers presenting with another primary breast cancer are extremely rare. In this article, we report on a 70-year-old male patient with bilateral multifocal and synchronous breast cancer and without a family history of breast cancer.

## Summary

Male breast cancer represents 1% of all breast cancers. It occasionally occurs in patients with a family history of this disease. Synchronous bilateral breast cancer in men is extremely rare. In this report, a 70-year-old male with synchronous bilateral multifocal cancer of the left breast (LB) with no family history of breast cancer is presented.

## Introduction

Breast cancer is a rare disease in men. According to international statistics, this pathology represents 1% of all breast neoplasia, whereas in Cuba, it represents 0.8% [1, 2].

Among the factors that predispose its occurrence, we include exposure to ionising radiation, the administration of oestrogens, and diseases related to hyperestroginisation, such as cirrhosis of the liver and Klinefelter syndrome [3].

Genetic factors such as the mutation of the BRCA1-2 genes or kinship are related to the incidence of this disease, although the majority of those affected do not present risk factors [4, 5].

Unlike its incidence in women, who have a bimodal age–frequency distribution, its incidence in men is unimodal, with a peak incidence at 71 years. It has a central, sub-areolar location, and nearly 85% of cases show up in a physical exam. The most common histological type is invasive (infiltrating) ductal carcinoma with an incidence of 85–90% [6–8].

Although the disease presents similarities in both genders, its rarity and the small number of cases do not allow the carrying out of clinical research necessary to develop optimum treatment. This is why treatment is based on empirical advances made in breast cancer in women [9].

In this report, we present a 70-year-old man with bilateral breast cancer, apparently synchronous, multifocal, and with differing histologies in the LB, with no family history of breast cancer. In this case, the clinical criteria for hereditary familial cancer are evaluated [1].

High risk

– Cancer at less than or equal to 40 years.
– Diagnosis of breast and ovarian cancer in the same individual.
– Two or more cases, one of which is less than 50 years old or bilateral.
– A case of breast cancer, less than or equal to 50 years and a case of ovarian cancer in a first- or second-degree relative.
– Three cases of breast and ovarian cancer in first- and second-degree relatives.
– Male breast cancer and breast or ovarian cancer in first- or second-degree relatives.

Moderate risk

– Two first-degree relatives if both have been diagnosed between the ages of 51 and 60.
– A first- and second-degree relative (mother or sister, and maternal aunt or grandmother), if the sum of their ages is less than or equal to 118 years.

## Clinical case

This is a 70-year-old white male with a history of type II diabetes mellitus. The patient denied any family history of breast or any other type of cancer. This information comes from a genetic and breast cancer consultation, in which the family history, physical examination, and mammography were carried out on the first-degree relatives of the patient. The patient came to the Oncology Institute complaining of lumps in both breasts for the past two months.

**Figure 1.**
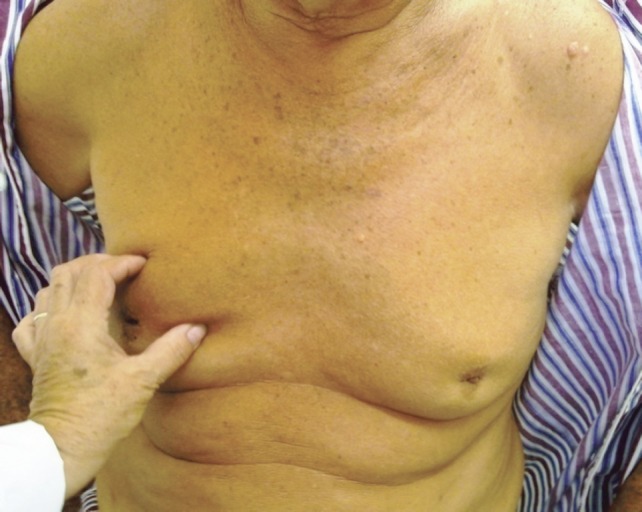
Patient aged 70 years with synchronous bilateral breast cancer.

Upon physical examination, a hard lump, measuring 30 x 20 mm, was felt in the right breast (RB) and armpit, with an irregular surface, poorly defined edges, mobile, with fixation and retraction of the nipple–areolar complex accompanied by hard, mobile, and ipsilateral adenopathy. The LB presented a hard, irregular lump, measuring 20 x 25 mm, with poorly defined edges in the retroareolar region with hard, mobile, and ipsilateral adenopathy (Figure 1).

**Figure 2.**
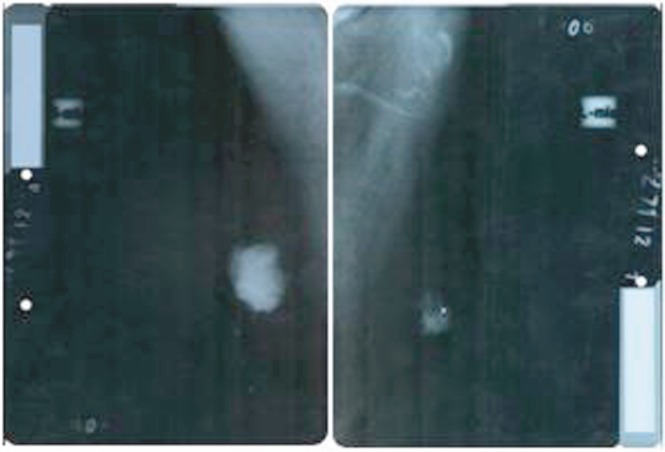
Bilateral mammogram: mediolateral oblique view showing the presence of lesions highly suspicious for malignancy, BI-RADS category V.

On performing the breast ultrasound, a solid, irregular lump was observed in RB, measuring 30 x 25 mm, of heterogeneous texture, with microcalcifications in its interior and adenopathies with a pathological appearance, while in the LB, a solid lump was observed, with a heterogeneous texture, measuring 22 x 20 mm, with irregular contours, located in the retroareolar region with no evidence of pathological adenopathies. The bilateral mammogram revealed the presence of a lump in the RB, with diffuse edges, infiltrates, measuring 40 x 35 mm, producing retraction and flattening of the nipple, with pathological microcalcifications in its interior, the spikes of the lump were close to the pectoralis major muscle, while in the LB, a lump was observed measuring 25 x 20 mm with diffuse spiked edges, with pathological microcalcifications both within and outside it (Figure 2). Both tests were classified, according to the Breast Imaging-Reporting and Data System (BI-RADS), as a category V lesion: highly suggestive for malignancy.

A freeze biopsy was performed, and invasive papillary carcinoma was reported for both breasts. A bilateral modified radical mastectomy was carried out.

**Figure 3.**
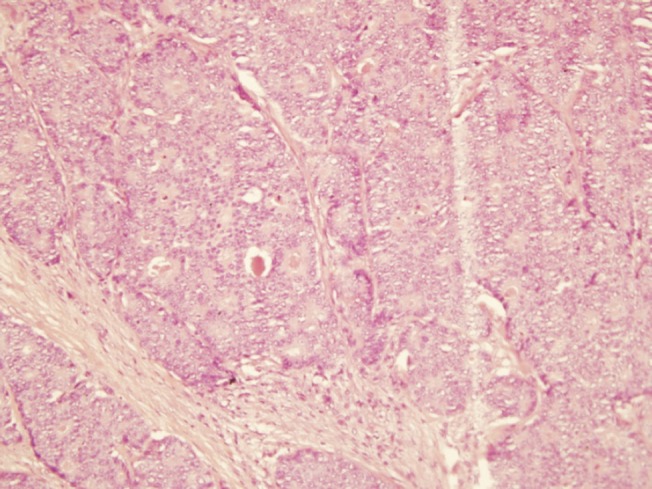
Haematoxylin-Eosin stain (20x). B12-2165: RB—invasive ductal carcinoma of intermediate-grade malignancy (NGII, BRII), measuring 4.5 x 4 x 3 cm.

**Figure 4.**
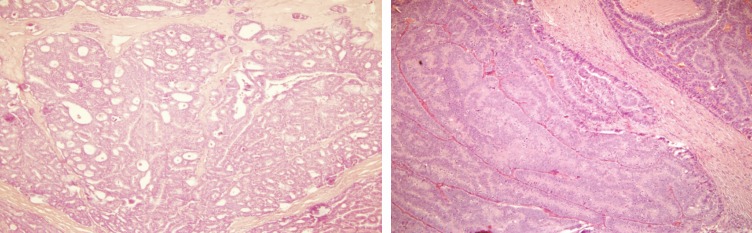
Haematoxylin-Eosin stain (20x). B12-2166: LB—invasive multifocal ductal carcinoma of intermediate-grade malignancy (NGII, BRII), with papillary and cribriform features, measuring 23 x 20 x 15 cm.

**Figure 5.**
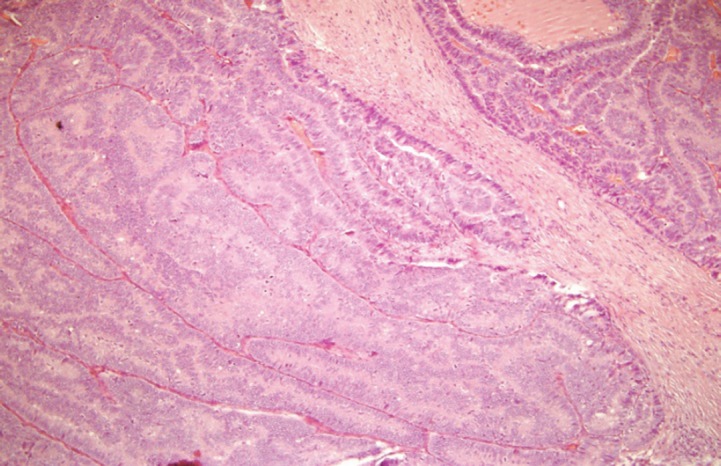
Haematoxylin-Eosin stain (20x). B12-2166: LB—invasive papillary carcinoma measuring 25 x 15 x 15 mm.

The histopathological examination revealed the presence of invasive ductal carcinoma in the RB, nuclear grade (NG) II, Bloms Richardson (BR) II, RE 45%, RP 85%, HER-2 +1, KI 67 40% with vascular and lymphatic invasion, and a tumour size of 45 x 40 x 30 mm. There were 11 positive nodes of a total of 20 analysed (11 of 20). In the LB, two (multifocal) lumps are reported, the larger measuring 23 x 20 x 15 mm corresponding to invasive ductal carcinoma, NG II, BR-II, RE 90%, RP 90%, HER-2 +1, KI 67 20%, with papillary areas, infiltration of adipose tissue, neural invasion, and vascular and lymphatic permeation. The other lump corresponds to invasive papillary carcinoma, measuring 25 x 15 x 15 mm and presented multiple calcifications. The lymph nodes examined showed two positive lymph nodes (2 of 18) (Figures 3–5). The chest x-ray, abdominal ultrasound, and bone scan carried out to assess the extent of the disease were within the norm.

According to the pTNM classification, this corresponds to pT2N3M0, stage IIIC for RB and pT2N1M0, stage IIB for LB. The patient was assessed in a multidisciplinary consultation where chemotherapy treatment was decided upon: four cycles of Adriamycin/Cyclophosphamide and four cycles of Taxol, in addition to radiation therapy to both surgical beds and supraclavicular fossa, followed by hormone therapy (one tablet of Tamoxifen daily for five years). At the present time, the patient’s condition is under control, and he is in follow-up with mastology.

## Discussion

Breast cancer in men is a rare pathology. The median age at diagnosis is 60–70 years, although it can affect men of all ages. A bilateral, synchronous presentation is extremely rare, with an incidence of 1–2.5% of the total number of patients with breast cancer [5, 10–13].

Bilateral breast cancer is defined as the presence of an independent primary malignant tumour in each mammary gland; while the term ‘synchronous’ refers to the presence of primary tumours in both breasts, which are diagnosed simultaneously. According to McCredie, this term also includes the diagnosis of contralateral tumours that occur within the first six months following diagnosis of the primary tumour, and according to Heron, within the first year of the initial diagnosis [10, 15, 16].

The most frequent histological type in men is the invasive ductal carcinoma (85–90%). Other rarer invasive tumours are invasive (4.5%) and mucinous (2.8%) papillary carcinoma. Carcinoma *in situ *has a frequency of 10% and is mostly papillary (74%) with a normally cystic presentation. Invasive papillary carcinoma is twice as frequent in men as it is in women (2–4% versus 1%) [17, 18].

With respect to the involvement of the lymph nodes in breast carcinoma in men, it has been observed that the more advanced the average age at diagnosis, the more likely that there will be lymph node involvement presenting at a more advanced stage [8].

In males, these tumours show a large oestrogen receptor (80–90%) and progesterone receptor (73–81%) expression, even higher than in women (75% and 65%, respectively). Some studies have shown a lower HER-2 expression in men (2–15%) than in women (18–20%), although the data are inconsistent [19, 20].

Due to the rarity of the disease, there are no controlled prospective studies that support a specific therapeutic management strategy. Therefore, almost all the management strategies in men are the result of retrospective studies of a series of cases and experience with women. The mainstay of treatment is based on the local and regional control of the disease with surgery and radiation therapy and in systemic control with hormone therapy and chemotherapy [21].

In the case of our patient, the histological types found correspond to those reported in the literature as the most frequent: papillary and invasive (infiltrating) ductal carcinoma, with multifocality with different histological types being truly rare [22]. The result of the studies of immunohistochemistry coincides with the reviewed literature, these highly endocrine tumours being responsive without overexpression of the HER-2 [23, 24]. Neither a family history of this pathology nor other risk factors associated with the disease were collected, so this is considered a sporadic and unusual case.

## Conclusion

We are dealing with an uncommon presentation of breast cancer in men: synchronous, bilateral, and multifocal with different histological types in the LB, with no family history of the pathology, or related risk factors, which is why its publication is considered important.
